# Evaluation of host and viral factors associated with severe dengue based on the 2009 WHO classification

**DOI:** 10.1186/s13071-014-0590-7

**Published:** 2014-12-11

**Authors:** Jorge O Pozo-Aguilar, Verónica Monroy-Martínez, Daniel Díaz, Jacqueline Barrios-Palacios, Celso Ramos, Armando Ulloa-García, Janet García-Pillado, Blanca H Ruiz-Ordaz

**Affiliations:** Departamento de Biología Molecular y Biotecnología, Instituto de Investigaciones Biomédicas, Universidad Nacional Autónoma de México (UNAM), Código Postal 04510 México, DF México; Departamento de Biología Celular y Fisiología, Instituto de Investigaciones Biomédicas, UNAM, Sede del tercer Circuito Exterior, México, DF México; Centro de Investigación sobre Enfermedades Infecciosas, Instituto Nacional de Salud Pública (INSP), Av. Universidad 655, Col. Santa María Ahuacatitlán 62508, Cuernavaca, Morelos México; Centro Regional de Investigación en Salud Pública, INSP. 4ª. Norte esquina con 19 Poniente, Código Postal 30700 Tapachula, Chiapas México

**Keywords:** Dengue virus, Severe dengue, 1997 WHO dengue case classification, 2009 WHO case classification, Risk factors, Viral load, Secondary infection

## Abstract

**Background:**

Dengue fever (DF) is the most prevalent arthropod-borne viral disease affecting humans. The World Health Organization (WHO) proposed a revised classification in 2009 to enable the more effective identification of cases of severe dengue (SD). This was designed primarily as a clinical tool, but it also enables cases of SD to be differentiated into three specific subcategories (severe vascular leakage, severe bleeding, and severe organ dysfunction). However, no study has addressed whether this classification has advantage in estimating factors associated with the progression of disease severity or dengue pathogenesis. We evaluate in a dengue outbreak associated risk factors that could contribute to the development of SD according to the 2009 WHO classification.

**Methods:**

A prospective cross-sectional study was performed during an epidemic of dengue in 2009 in Chiapas, Mexico. Data were analyzed for host and viral factors associated with dengue cases, using the 1997 and 2009 WHO classifications. The cost–benefit ratio (CBR) was also estimated.

**Results:**

The sensitivity in the 1997 WHO classification for determining SD was 75%, and the specificity was 97.7%. For the 2009 scheme, these were 100% and 81.1%, respectively. The 2009 classification showed a higher benefit (537%) with a lower cost (10.2%) than the 1997 WHO scheme. A secondary antibody response was strongly associated with SD. Early viral load was higher in cases of SD than in those with DF. Logistic regression analysis identified predictive SD factors (secondary infection, disease phase, viral load) within the 2009 classification. However, within the 1997 scheme it was not possible to differentiate risk factors between DF and dengue hemorrhagic fever or dengue shock syndrome. The critical clinical stage for determining SD progression was the transition from fever to defervescence in which plasma leakage can occur.

**Conclusions:**

The clinical phenotype of SD is influenced by the host (secondary response) and viral factors (viral load). The 2009 WHO classification showed greater sensitivity to identify SD in real time. Timely identification of SD enables accurate early decisions, allowing proper management of health resources for the benefit of patients at risk for SD. This is possible based on the 2009 WHO classification.

## Background

Dengue is a systemic and acute viral disease caused by the four serotypes of the dengue virus (DENV), transmitted to humans through the bites of infected *Aedes* mosquitoes, principally *A. aegypti* and *A. albopictus*. This mosquito is a tropical and subtropical species widely distributed around the world, mostly between latitudes 35°N and 35°S [1,2]. Dengue fever (DF) is the most prevalent of vector-borne diseases. According to the World Health Organization, about 100 million cases occur annually worldwide, with a mortality rate of 2.5% and more than 3 billion people live in dengue-endemic regions [3]. Moreover, the global impact of dengue has been recently estimated to be 390 million infections per year [4]. The recent increase in dengue case numbers is associated with the continuing dispersion of both DENV and mosquito vectors to new geographic regions [3]. DF presents a broad clinical spectrum, ranging from a benign self-limiting infection (85–90% of cases) to the most severe forms (approximately 10–15% of cases) such as dengue shock syndrome (DSS) and dengue hemorrhagic fever (DHF) [5]. Currently, the most severe cases do not always fit the four strict criteria (fever, hemorrhage, thrombocytopenia, and plasma leakage) of the 1997 WHO disease classification into DF or DHF/DSS. The WHO in 2009 improved the dengue case classification based on clinical severity, and this has been included in the WHO 2009 guidelines [1] and therefore it would be better to use this as a reference. The process has been described by Horstick *et al.* [6]. Although the 2009 WHO classification was designed primarily for use as a clinical tool, it also enables cases of SD to be differentiated into three specific subcategories; Severe vascular leakage, severe bleeding, and severe organ dysfunction, that could allow clinicians to evaluate the severe disease progression or pathogenesis in a more focused way, providing a new framework for scientific research [7]. It is known that host and viral factors play a role in the development of more severe dengue cases [8-10]. Two main hypotheses have been proposed to explain these epidemiological observations. First, the antibody-dependent enhancement hypothesis suggests that severe disease occur along with secondary infections when antibodies from a primary infection with a different serotype enhance the binding of heterologous IgG–DENV complexes to Fcγ receptors on macrophages, amplifying the infection; the increased viral load then leads to an immunopathogenic response [8]. The virulence hypothesis suggests that some DENV strains are more virulent than others, leading to a more severe disease [9]. The infecting serotype or genotype also influences disease severity [11-13]. A positive correlation between viremia and disease severity has been demonstrated, supporting both hypotheses [14,15]. The aim of the present study was to evaluate the host and viral factors that could play a role in the progression of severe dengue cases in the frame of the revised WHO classification. As well, no previous study has applied the 2009 WHO scheme to Mexico, where dengue fever is endemic and is reported in 28 out of 32 states. We present evidence on the association of risk factors with SD based on the 2009 WHO classification.

## Methods

### Study area and study population

A prospective cross-sectional study was carried out during an epidemic of dengue in 2009 in the central region of the State of Chiapas, Mexico. Patients with diagnoses of probable DF, DHF or DSS were admitted to public hospitals (secondary and tertiary level) or health centers (primary care level) in Chiapas, Mexico. Clinical, epidemiological and laboratory data were registered prospectively in the Health Department’s official forms used for the study of dengue cases. Patients who showed clinical or laboratory evidence of other diseases were excluded, as in 2009 the AH1N1 influenza epidemic and the dengue epidemic occurred simultaneously.

### Ethical considerations

Informed consent was obtained to participate in the study from each patient or patient’s parents (in the case of children) after a full explanation of the study. The study protocol was reviewed and approved by health authorities in Chiapas, Mexico.

### Dengue case classification

Between June and October 2009, a cohort of 630 patients was enrolled. Dengue cases were initially classified according to the 1997 WHO criteria [16] to comply with Mexican public health standards [14]. However, all cases were also simultaneously classified using 2009 WHO criteria. The sensitivity and specificity for the identification of disease severity were evaluated using both classifications. For the D/SD case classification, we applied 2009 WHO criteria. D–WS was defined as fever with two or more of the following criteria: pain (myalgia, arthralgia, headache), nausea, vomiting, positive tourniquet test or exanthema. Patients with D + WS showed abdominal pain, persistent vomiting, mucosal bleeding, lethargy, restlessness, hepatomegaly >2 cm or an increase in hematocrit concurrent with a rapid decrease in platelet count. SD was defined by one or more of the following manifestations: plasma leakage (hemoconcentration ≥20%) that could contribute to the presence of hypovolemic shock or fluid accumulation; severe bleeding and/or severe damage to organs such as the liver (aspartate aminotransferase or alanine aminotransferase levels ≥1000 IU/μL), heart, central nervous system or other organs [1]. Likewise, for evaluating the sensitivity and specificity of both classifications, the level of clinical care that the patient required for his/her treatment was taken into account [15,17]. Level I included outpatients (who did not require admission to hospital); level II included inpatients that received intravenous fluids for rehydration or maintenance; level III included patients admitted to an intensive care unit (ICU) who received oxygen therapy, inotropic agents or hemocomponents. For both classifications, the cost–benefit ratio (CBR) was evaluated. Benefit (B) was defined as the increase in sensitivity to detection of SD cases. In other words, B was defined by the quotient of SD cases detected according to the 2009 classification criteria divided by DHF grade III–IV cases detected according to the 1997 WHO classification criteria. Cost (C) was defined as the overloading on health units caused by patients that required admission to hospital for their treatment. In other words, C was defined by the quotient of D + WS cases plus the cases of SD according to the 2009 classification, divided by DHF cases detected according to the 1997 classification criteria; thus, CBR = C/B.

### Dengue diagnosis and laboratory tests

Dengue diagnosis was confirmed with the NS1 antigen detection test using a commercial kit (Platelia Dengue NS1 Ag, Bio-Rad Laboratories, Marnes La Coquette, France), or by detecting anti-dengue IgM or IgC antibodies using an enzyme*-*linked immunosorbent assay (ELISA) (Panbio, Brisbane, Australia). Patients with negative results were excluded from this study. Platelet counts and hematocrits were determined every 24 h.

### Antibody response

To evaluate the antibody response, the sample size was calculated utilizing the formula for finite populations proposed by Daniel [18]. To assess the response type (primary and secondary) we utilized the IgG avidity test (Dengue ELISA IgG kit, Focus Technologies, Cypress, CA, USA) according to De Souza et al. [19].

### Dengue RNA detection and typing

Viral RNA extraction and purification (140 μL of serum) were done in duplicate utilizing the QIAamp Viral RNA kit (QIAGEN, Hilden, Germany) following the manufacturer’s instructions [20]. DENV typing was carried out by end-point polymerase chain reaction (PCR) assay, utilizing RNA purified from serum samples as described [21]. Assays were conducted in a GeneAmp PCR System 2400 thermal cycler (Applied Biosystems, Foster City, CA, USA). To optimize the second amplification reaction, the original protocol was slightly modified (with a second amplification reaction with 3 μL of a 1:10 dilution of the product from the initial amplification reaction) utilizing the following conditions: 35 denaturalization cycles (94°C, 30 s), primer blend (54°C, 40 s) and extension (72°C, 40 s).

### Dengue viremia quantification by real-time reverse-transcriptase polymerase chain reaction (RT–PCR)

A real-time RT–PCR assay was used to quantify the viral genome concentration (StepOne; Applied Biosystems). The primers and probe (designed by our group and synthesized by Custom TaqMan Gene Expression (Applied Biosystems, ID 186606936) were added to 5 μL of purified RNA (in duplicate) and added to the master mix of One-Step RT–PCR Master-Mix Reagents kit (Applied Biosystems) in a final volume of 20 μL. For sample analysis, in the StepOne RT–PCR system (Applied Biosystems), a retrotranscription reaction was carried out before the real-time PCR, which allowed the synthesis of complementary DNA (cDNA) from viral RNA, using the Multiscribe Reverse Transcriptase enzyme recombinant of Moloney Murine Leukemia Virus (rMoMuLV). The cDNA was then used as a template for amplification of the product by real-time PCR.

### Data analysis

Means and standard deviations were determined for the statistical analysis of quantitative variables, while frequencies of each category were calculated for qualitative variables. For comparison of groups, we used Fisher’s exact test and calculated the odds ratio (OR) and 95% confidence interval (CI). Means between two or three groups were compared using the Mann–Whitney nonparametric *U* test, the Kruskal–Wallis test or Dunn’s multiple comparison test; p < 0.05 was considered statistically significant. A receiver operating characteristics curve (ROC) was utilized to assess the sensitivity and specificity of platelet counts to identify cases of SD. In addition, a logistics regression analysis was conducted to determine risk factors associated with the probability to discriminate cases of SD from D ± WS, according to the 2009 WHO classification. For logistic regression, variables analyzed were previously selected from the correspondence analysis: (a) disease phase (toxic or defervescence); (b) type of infection (primary or secondary); (c) level of viremia (low, ≤3.5; or high, >3.5 log_10_ copies/μL); and d) platelet count (low, ≤35,000/μL; or high, >35,000/μL). Goodness of fit of the model was made using the statistical analysis of deviation and Pearson’s correlation; in both cases, the existence of deviation (p > 0.05) was rejected. The effect of predicting variables was taken as significant when p < 0.05 according to Wald’s test for type III effects. Correspondence analysis and logistic regression were carried out utilizing the Proc Logistic module included in the SAS package version 9.0 (SAS, Cary, SC, USA).

## Results

### Epidemiological characteristics of the study population

Of the 630 cases engaged in the study, 141 (23.4%) had negative serological tests for DENV and were excluded. The remaining 489 samples (77.6%) had positive serological tests for the NS1 DENV antigen and IgM, or IgG response. According to the calculation of sample size for the purpose of the different experiments, 220 samples were selected: 76 of SD and 144 of D ± WS. The age range of patients was 0–79 years (mean = 25, standard deviation ± 15). The age of 30.9% of the study population was ≤15 years, showing that dengue affect both children and adults. These results are similar to those reported in Brazil, where there is a similar infection ratio (26.6%) in children and young adults [22]. These results show a transition in the epidemiological behavior of dengue in the Americas from being a disease that occurs in childhood to one affecting all age groups. Table 1 shows that SD was significantly more frequent than D ± WS in the age group of 9–16 years, according to Fisher’s exact test (p = 0.0341, OR, 1.661; 95% CI, 1.043–2.645). Of the total population (489), 236 patients (48.3%) were female and 253 (51.7%) were male (Table 2). This gender ratio was similar according to Fisher’s exact test (p = 0.5222). We found that DENV-2 was the predominant serotype (99%); however, DENV-1 was also detected. A secondary antibody response was present in 85.6% of patients. When DENV infection severity was analyzed, 70 patients with SD (97%) and 119 (80%) with D ± WS showed a secondary antibody response. These data indicate a strong association between secondary antibody response and the development of SD (OR, 8.5; 95% CI, 1.9–36.8). These results are consistent with previous studies using the 1997 WHO (DF/DHF/DSS) scheme, carried out in Cuba [23], Thailand [24], Burma [25] and Vietnam [26-28]. By applying the 2009 WHO classification, all cases of DHF (grades III–IV) remained in the SD group, and the grade of DF was strongly associated with the likelihood of D–WS.

**Table 1:** **Population distribution by age group**

**Age group (years)**	**Non-severe dengue**		**Severe dengue**		**TOTAL**	
	**n**	**%**	**n**	**%**	**n**	**%**
0–8	32	8.56	12	10.43	44	9.00
9–16**	83	22.19	40	34.78	123	25.15
17–24	100	26.74	29	25.22	129	26.38
25–32	46	12.30	18	15.65	64	13.09
33–40	41	10.96	11	9.57	52	10.63
41–48	26	6.95	3	2.61	29	5.93
49–56	24	6.42	1	0.87	25	5.11
57–64	14	3.74	0	0.00	14	2.86
65–72	7	1.87	1	0.87	8	1.64
73–80	1	0.27	0	0.00	1	0.20
Total	374	100.00%	115	100.00	489	100.00

*P* = 0.0096, Fisher’s exact test. **The age group 9–16 is significantly more susceptible than the rest of the population to present the severe form of the disease.

**Table 2:** **Distribution of cases by gender**

**Gender**	**Severe dengue**		**Non-severe dengue**		**TOTAL**	
	**n**	**%**	**N**	**%**	**n**	**%**
Male	63	54.8	190	50.8	253	51.7
Female	52	45.2	184	49.2	236	48.3
Total	115	100	374	100.00	489	100

*P* = 0.5222, Fisher’s exact test. There are no significant differences between genders in relation to presentation of the severe form of the disease.

**Figure 1:**
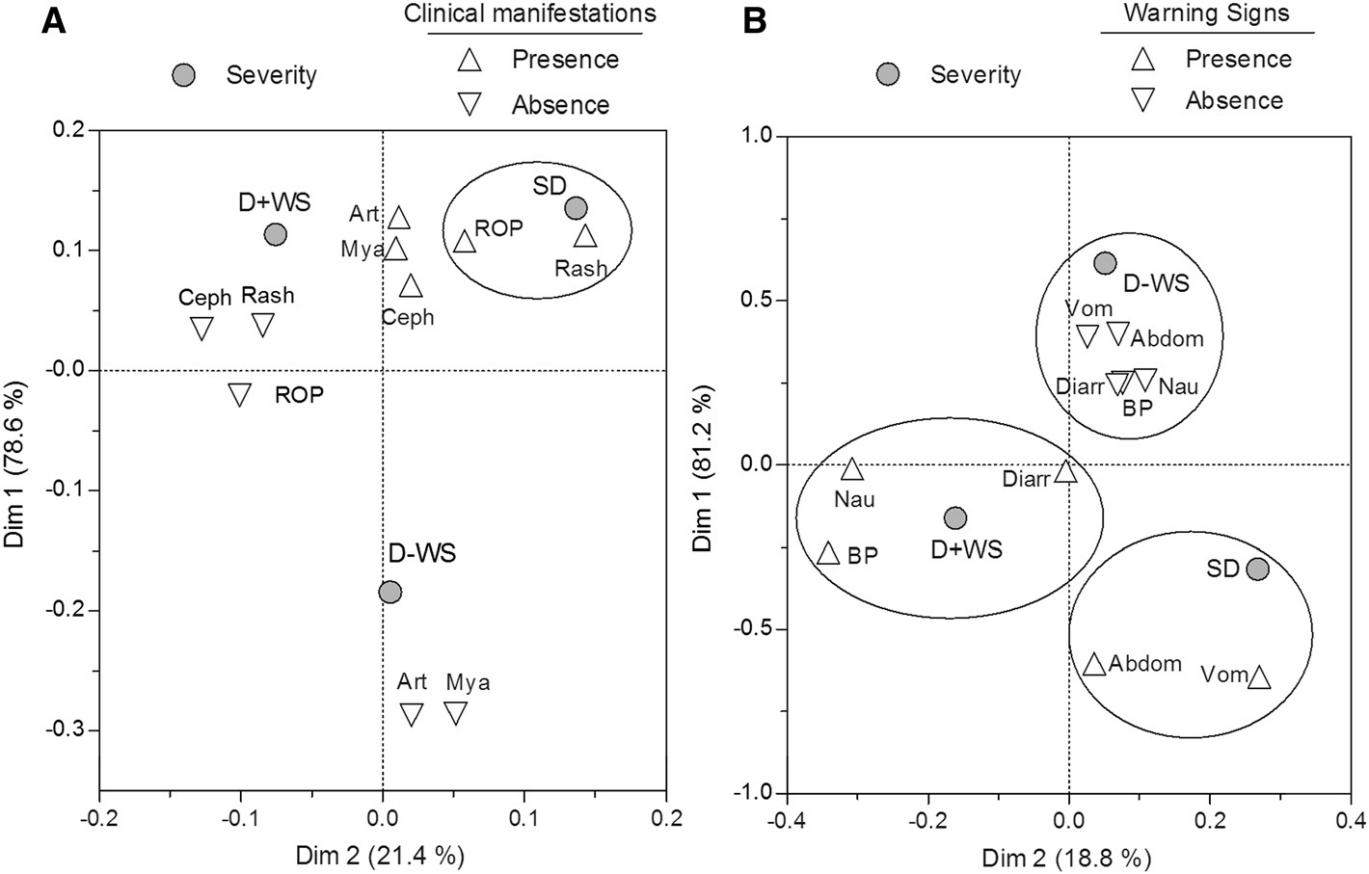
**Correspondence analysis between clinical manifestations and severity of illness. (A)** Correspondences between dengue severity and main clinical manifestations are shown. SD showed a close correspondence with rash and retro-orbital pain (ROP). **(B)** Correspondences between dengue severity and warnings signs are shown. A closer correspondence between SD and vomiting and abdominal pain was observed. Diarrhoea exhibited an equal correspondence with SD and D ± WS. D–WS was distinguished by a close correspondence with an absence of vomiting, abdominal pain, diarrhoea, nausea, and back pain (BP).

**Figure 2:**
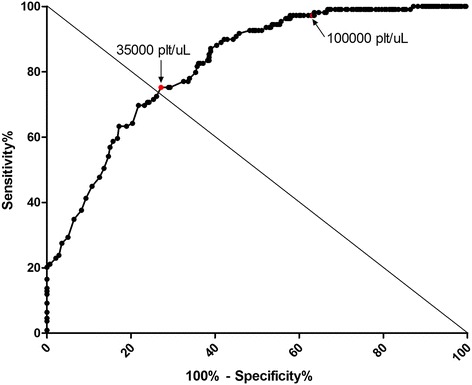
**Sensitivity (S) and specificity (E) of platelet count for prediction of severe dengue.** Several intersection (cut-off) points were selected and S and E calculated for each. On the coordinates (abscissas) axis (1-E) and S on the ordinates axis. The ideal examination (S = 1 and E = 1) should be on the top-left angle of the graph. Cut-off value of 35,000 plat/*μ*L showed the greatest sensitivity and specificity (S = 75% and E = 72.86%). A cut-off value of 100,000 plat/*μ*L showed S of 97.25% and E of 37%. (N = 389, area under the curve ROC = 0.8199).

The most frequent clinical manifestations in both the SD and D ± WS cases were myalgia, arthralgia, and headache, which did not show statistical significance to differentiate SD from D ± WS. Surprisingly, exanthema and retro-orbital pain showed a high correspondence with SD as confirmed by Fisher’s exact test (Table 3). D–WS was highly associated with the absence of persistent vomiting, abdominal pain, diarrhea, nausea or lumbosacral pain, arthralgia and/or myalgia (Figure 1A). With regard to warning signs (WS) proposed in the 2009 WHO scheme, we observed a high correspondence between persistent vomiting, abdominal pain, mucosal bleeding and SD (Figure 1B). These results were confirmed by Fisher’s exact test, in which persistent vomiting, abdominal pain, petechiae, epistaxis, and gingival bleeding, showed significant differences between cases of SD and D ± WS (Table 3). In the SD group, 62 (56.9%) patients presented plasma leakage, 40 (36.7%) presented bleeding, and four (3.7%) hepatomegaly. In these patients, the following bleeding disorders were presented: hematemesis in 23 cases (21.1%); melena in 11 cases (10.1%); and metrorrhagia in eight (7.3%). None of these disorders was found in patients with (D ± WS). The average platelet count nadir in the SD group (21,000/μL) was significantly lower than in the D ± WS group (66,000/μL; p < 0.0001 by Mann–Whitney test). The sensitivity and specificity of platelet count for association with SD were assessed (Figure 2). From ROC curves, several thresholds were selected for determining each value. A threshold of 35,000/μL showed a more balanced sensitivity (S) and specificity (E) (S = 75% and E = 72.86%; N = 389, area under ROC curve = 0.8199; 95% CI, 0.7767–0.8631). A threshold of 100,000/μL (necessary criterion for DHF) showed an elevated S (97.25%) but a low E (37%). Among WS proposed by the revised WHO classification, we found support for abdominal pain, vomiting and mucosal bleeding. As was defined by the warning signs in the 2009 WHO scheme, which were considered to require more specific definitions [29]. Currently, two large global prospective studies are underway to help improve the knowledge of the value of WS: The International Research Consortium on Dengue Risk Assessment Management and Surveillance Study (IDAMS; http://ichgcp.net/clinical-trials-registry/NCT01550016) and also the Laboratory Diagnosis and Prognosis of Severe Dengue Study at http://ichgcp.net/clinical-trials-registry/NCT01421732.

**Table 3:** **Clinical manifestations associated with dengue**

**Clinical signs and symptoms**	**D ± WS** ***n*** **= 380 (%)**	**SD** ***n*** **= 109 (%)**	***P*** **value** ^**1**^
***Frequent signs and symptoms***			
**Cephalea**	360 (94.7)	105 (96.3)	
**Myalgia**	346 (91.0)	102 (93.5)	
**Arthralgia**	322 (84.7)	98 (89.1)	
**Rash**	151 (39.7)	58 (53.2)	<0.05
**Retro-orbital pain**	264 (69.4)	86 (78.9)	<0.05
**Nausea**	45 (11.8)	7 (6.4)	
**Diarrhoea**	21 (5.5)	7 (6.4)	
***Warning signs***			
**Abdominal pain**	50 (13.1)	32 (29.3)	<0.001
**Vomiting**	39 (10.2)	37 (33.9)	<0.001
**Back pain**	14 (3.6)	3 (2.7)	
**Epistaxis**	34 (8.9)	32 (29.3)	<0.05
**Gingivorrhagia**	35 (9.2)	21 (19.2)	<0.05
**Petechiae**	41 (10.7)	26 (23.8)	<0.05
**Equimosis**	3 (0.7)	5 (4.5)	

^1^ *P* values were calculated using Fisher’s exact test. Only significant differences among groups are shown.

### Evaluation of the 1997 and 2009 WHO classifications with regard to the level of clinical care required

**Figure 3:**
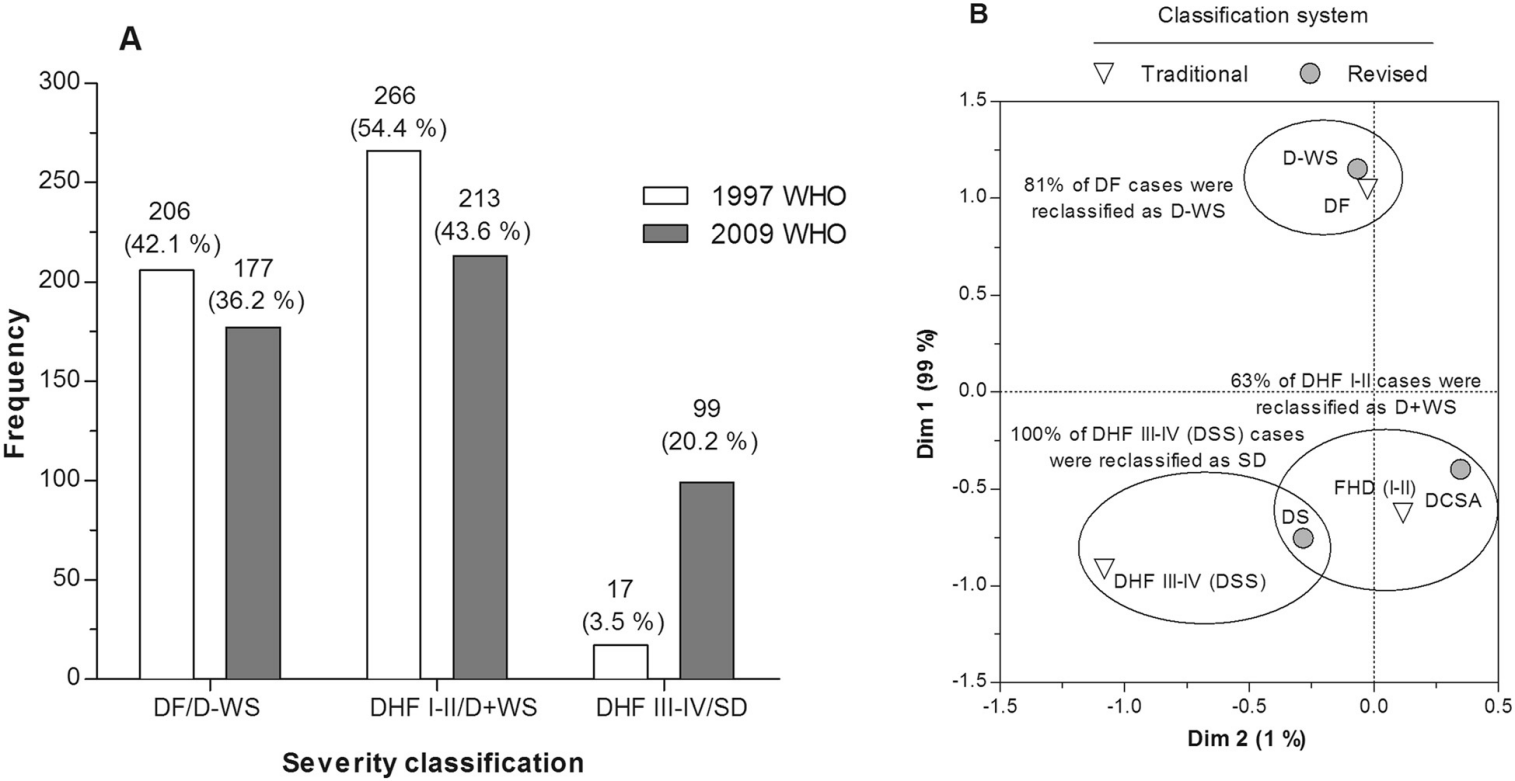
**Evaluation of the 1997 and 2009 WHO classifications to identify SD. (A)** The percentage of laboratory-confirmed dengue cases classified as DF, DHF, or DSS in the traditional scheme (1997 WHO), or classified as D–WS, D + WS, or SD according to the 2009 WHO scheme is shown. **(B)** Correspondence between traditional and revised classification.

According to the 1997 WHO classification, 206 (42.1%) dengue cases were categorized as DF, 266 (54.4%) as DHF and 17 (3.5%) as DSS (Figure 3A). In the 2009 WHO classification, 99 cases were detected as SD (20.2%; Figure 3A) and 390 cases were detected as D ± WS (79.8%), of which 177 (36.2%) were classified as D–WS and 213 (43.6%) as D + WS.

In the framework of the WHO 1997 scheme, 88.3% (182/206) of dengue cases were treated as outpatients. However, 11.7% (24/206) were admitted to hospital (inpatients) and required intravenous (i.v.) fluids for rehydration. Likewise, 93.2% (248/266) of DHF cases received i.v. fluids and 35.3% (6/17) of DSS cases were treated following level III care. According to these data, the sensitivity to detect severe cases was 75% (95% CI_,_ 34.9–96.8) with a specificity of 97.7% (95% CI, 95.9–98.8) (Table 4).

**Table 4:** **Comparison of the 1997 classification** ***vs*** **level of care**

**1997 classification**	**Clinical intervention level**			**Total**
	**Level I**	**Level II**	**Level III**	
DF	182	24	0	206
DHF	16	248	2	266
DSS	0	11	6	17
Total	198	283	8	489
Sensitivity 75 (34.9–96.8)		Specificity 97.7 (95.9–98.9)		

For the WHO 2009 scheme, 160/177 (90.4%) of D–WS cases were outpatients and 17/177 (9.6%) received level II care. Level III care was given to 8/99 (8.1%) of cases of SD. That the eight cases treated at level III had been classified as SD showed a high level of sensitivity (100%; 95% CI, 63.1–100), with a specificity of 81.1% (95% CI, 77.3–84.5) (Table 5). According to these data, here we found that the 2009 classification showed higher sensitivity than the 1997 classification to detect cases of SD. Additionally, the CBR was calculated in terms of benefit (detection of a larger number of cases of SD to prevent dengue-related deaths) in relation to the cost (overloading of medical units). Interestingly, when evaluating the CBR for both classification schemes, the 2009 classification presented a high benefit (537%) with a low cost (10.2%); the CBR was 0.019.

**Table 5:** **Comparison of the 2009 classification vs level of care**

**2009 classification**	**Clinical intervention level**			**Total**
	**Level I**	**Level II**	**Level III**	
D–WS	160	17	0	177
D + WS	38	175	0	213
SD	0	91	8	99
Total	198	283	8	489
Sensitivity 100 (63.1–100)		Specificity 81.1 (77.3–84.5)		

### Viral load determination according to day of illness

**Figure 4:**
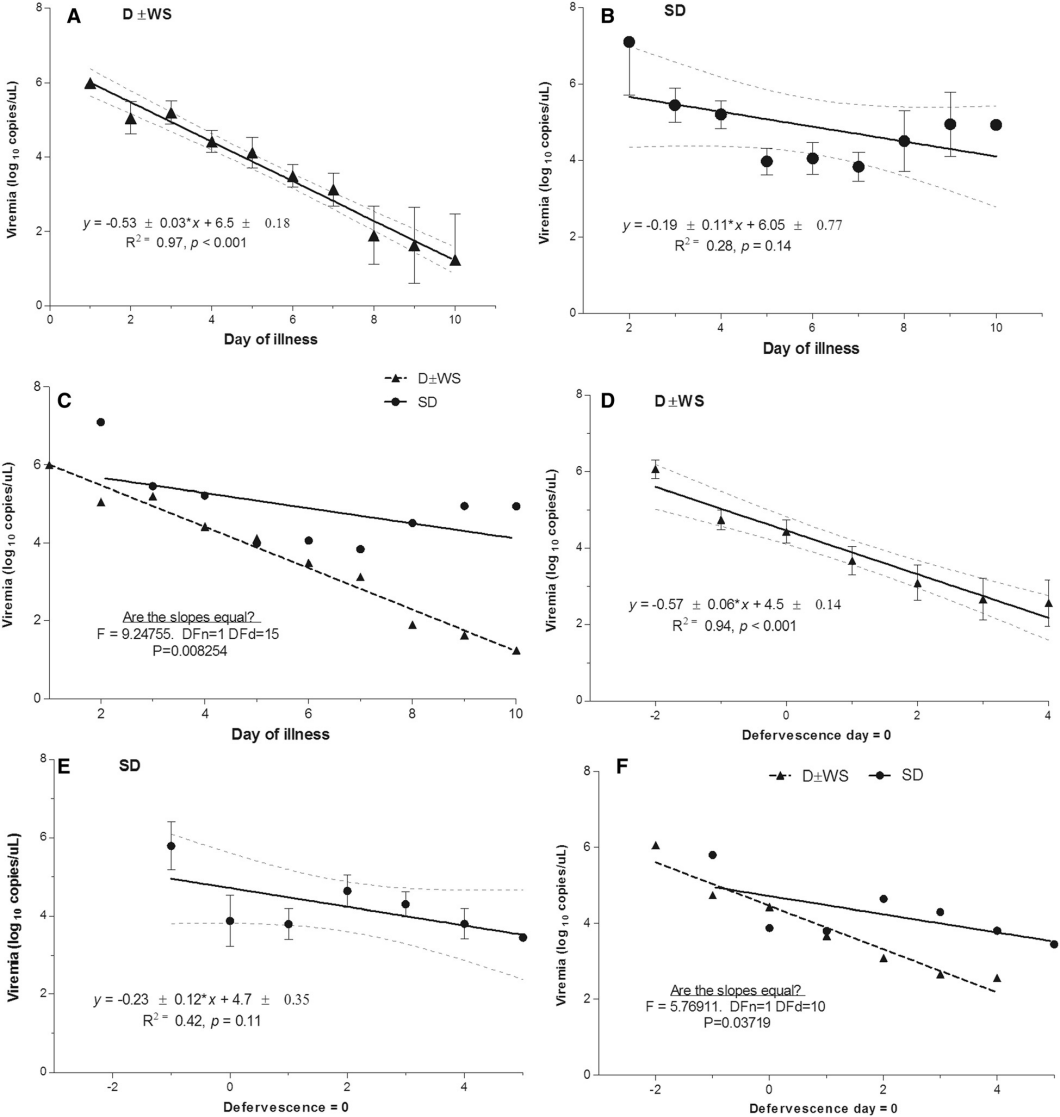
**Linear regression of viremia according to time. (A, C)** Viremia levels in D ± WS are explained by day of illness or defervescence day, respectively. **(B, D)** In contrast, linear regression can explain the viremia behavior in SD either according to illness day or according to defervescence day. **(E, F)** Slopes of viremia levels significantly differ between SD and D ± WS according to day of illness or defervescence day.

Viremia levels (medians) presenting on the day of illness (DOI) or on the day of defervescence for each category are shown in Figure 4A. In cases of D ± WS, viremia levels decreased significantly on the DOI (Pearson’s *r* = −0.9877, p < 0.0001). However, in SD cases, viremia did not drop in reference of the DOI (Figure 4B; Pearson’s *r* = −0.5314, *p* = 0.2824). The day of illness explained the 97.56% of the viremia tendency in cases of D ± WS. Likewise, kinetic changes in viremia in cases of SD and D ± WS were significantly different (p = 0.008254) according to the day of illness (Figure 4C).

### Viral load according to defervescence day

In cases of D ± WS, viremia dropped significantly during the defervescence stage (Figure 4D; *r* = −0.9711, p = 0.0003). The day of defervescence explained 94.31% of the viremia tendency in cases of D ± WS. However, in those patients with SD, high viremia levels persisted during defervescence (r = −0.6541, *P* = 0.1110; Figure 4E). Slopes of viremia in SD and D ± WS showed significantly different trends according to the defervescence stage (p = 0.03719; Figure 4F).

**Figure 5:**
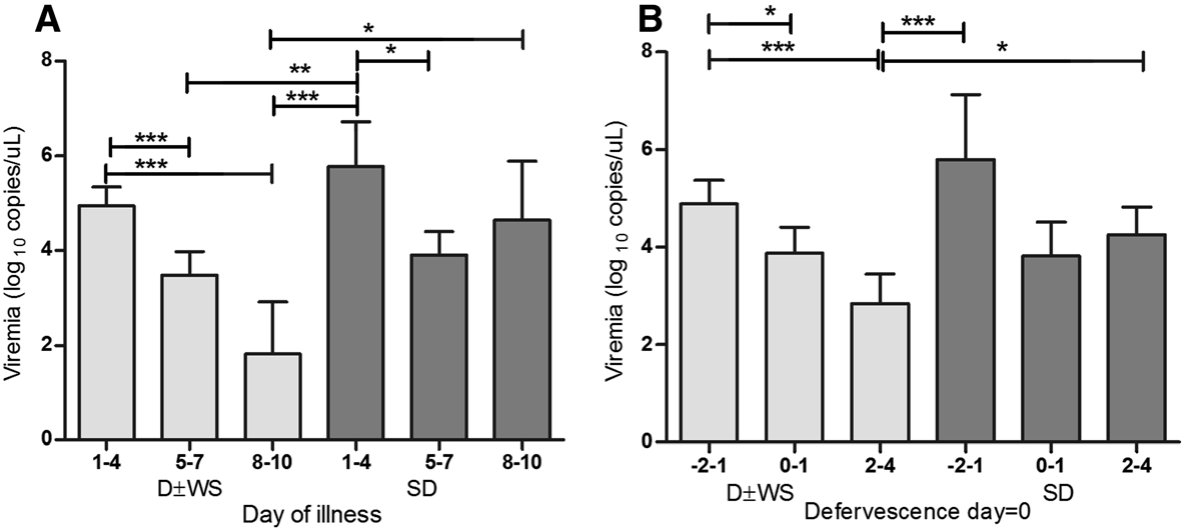
**Viremia levels during illness phase. (A)** Viremia levels of 219 D ± WS and SD patients from day 1 to day 10 of illness. During the first 4 days of illness, viremia levels in SD were higher than in D ± WS. In D ± WS, viremia levels significantly diminished over time. In contrast, in SD, viremia levels persistently rose. **(B)** According to defervescence, in the febrile stage, viremia levels were higher in SD than in D ± WS. During defervescence, viremia levels could still be observed in SD and in D ± WS. During defervescence days 2–4, viremia levels persistently rose in SD. In contrast, viremia levels diminished in D ± WS (Kruskal–Wallis test, multiple comparison Dunn’s *post hoc* test, *P* = 0.0001).

Simultaneously, we evaluated the viral load per stage (febrile, critical, or convalescent; see Methods). On DOI 1–4, viremia in cases of SD was higher than in those with D ± WS. Interestingly, in DOI 5–10, viremia in cases of SD remained elevated, whereas in those with D ± WS it decreased significantly (p < 0.0001; Kruskal–Wallis and Dunn’s multiple comparison tests; Figure 5A).

During the febrile stage, viremia in patients with SD was higher than in those with D ± WS. Interestingly, after defervescence, viremia in the former group remained high, whereas it decreased significantly in the latter (*p* < 0.0001 by Kruskal–Wallis test, and Dunn’s Multiple Comparison test; Figure 5B).

By logistic regression analysis four risk factors associated with a higher likelihood of presenting SD according to the 2009 WHO classification were detected (Table 5). Both viral load and illness stage were important variables for an association with SD. Patients who showed high viremia levels >3.5 Log_10_ copies/μL (OR, 3.36; 95% CI, 1.58–7.11) during the defervescence stage, presented a higher risk of SD (OR, 7.11; 95% CI, 2.41–20.9). Likewise, a platelet count of <35,000/μL proved a valuable variable for differentiating the severity of the disease. The SD patients showed a risk 2.84 times higher of those patients showing low platelet levels (Table 6).

**Table 6:** **Summary of logistic regression analysis to discriminate non-severe dengue patients from severe dengue patients according to the 2009 WHO classification**

**Variable**		**Confidence limits at 95%**	
	**Odds ratio**	**Inferior**	**Superior**
Illness phase: Defervescence or toxic *vs* febrile	7.11	2.41	20.9
Antibody response: Primary *vs* Secondary	5.67	1.22	26.22
Viremia level: <3.5 *vs* >3.5 (Log_10_ copies/*μ*L)	3.36	1.58	7.11
Platelet count: <35,000 *vs* >35,000/*μ*L	2.84	1.49	5.39

It was observed that patients who had secondary DENV infections were associated with a higher risk of having SD than patients with a primary infection (OR, 5.67; 95% CI, 1.22–26.22). Given that these four predictive variables were utilized together in risk analysis, it is important that such variables be considered together to increase the efficiency and strength of the prognosis on the disease severity.

Parallel, a logistic regression analysis was conducted to differentiate severe dengue cases (utilizing the same variables) following the 1997 WHO classification (Table 7). Contrary to data found in cases utilizing the 2009 WHO scheme, no risk factor was found (Table 7) to discriminate patients with DF from those with DHF stages I–II or from those with DHF stages III–IV (DSS), even though the model showed that illness stage, infection type, and platelet count had significant effects (p < 0.05). This finding might have been caused by the low incidence of patients (*n* = 17) in the highest level of severity (DHF grade III–IV) within such a classification. The difference in the ratio of severe cases might also be explained by the presence of 101 patients classified as having DF, because they did not strictly fulfill the 1997 classification criteria. These included 10 patients with a platelet count of ≤10,000/μL, two patients with severe bleeding (one with hemoconcentration), and 89 patients that did not show hypotension but showed hemoconcentration or severe bleeding. In addition, according to the 1997 classification, 283 cases identified as DHF/DSS required admission to hospital to monitor them closely. However, using the 2009 classification, 312 patients classified as having D + WS or SD required admission to hospital for close monitoring. Thus, there was an increase of only 10.2% (*n* = 29) in hospital admissions. There were 29 outpatient cases, and these individuals might have been at risk of developing severe illness.

**Table 7:** **Summary of logistic regression analysis to discriminate FD, FHD (I–II) patients from FHD (III–IV) patients according to the 1997 classification**

**Variable**	**Odds ratio**	**Confidence limits at 95%**	
		**Inferior**	**Superior**
Illness phase: Defervescence or toxic *vs* febrile	0.27	0.10	0.46
Antibody response: Primary *vs* secondary	0.11	0.03	0.32
Viremia level: <3.5 *vs* >3.5 (Log_10_ copies/*μ*L)	0.69	0.35	1.36
Platelet count: <35,000 *vs* >35,000/*μ*L	0.38	0.21	0.70

The logistic regression analysis explained the better sensitivity for identifying SD cases in the framework of the 2009 WHO classification.

## Discussion

Dengue is a complex illness in which a spectrum of the disease exists rather than two distinct entities (DF or DHF/DSS). With the improved description of dengue cases by the WHO in 2009 [1], a dengue categorization was established based on clinical severity. A good systematic literature review in its field was recently reported [30].The need for a clinically relevant case classification which is easy to apply is important, because the early identification and the opportune treatment of severe or potentially severe dengue cases ensures that limited resources are directed to those most in need. This critical situation arose during the simultaneous incidence of outbreaks of dengue and AH1N1 influenza epidemics in Chiapas, Mexico in 2009. Thus, it is important to identify cases of SD promptly to make accurate and early decisions, allowing for the suitable management of health-sector resources, and benefitting patients at risk of SD. This outcome was possible based on the 2009 WHO classification. For a more precise definition of clinical dengue phenotypes with epidemiological utility (also for dengue pathogenesis studies), it is necessary to obtain different epidemiological relevant data that allow the evaluation of host and viral factors in real time, as in the case of the 2009 WHO scheme.

We found that the 2009 WHO classification had a higher sensitivity to detect patients with SD, as it did not require or fulfill the strictly criteria of the 1997 WHO scheme, thus resulting in an identification of SD in real time. For such reasons, our corresponding analysis showed a medium to low level of agreement between both classification schemes (Kappa index = 0.54; 95% CI, 0.48–0.59; p < 0.001; Table 3).

We performed a logistic regression analysis (Table 6) for identifying risk factors associated with the likelihood of discriminating between patients with and without severe cases according to both WHO classifications. We used the four variables that were selected from the correspondence analysis: disease phase (toxic or defervescence); type of infection (primary or secondary); viremia load (low ≤3.5, or high >3.5 Log_10_ copies/μL); and platelet count (low ≤35,000, or high >35,000/μL). With these variables, we were able to identify those factors with a higher probability of discriminating between patients with and without SD. We found that secondary infections, disease phase and viral load were important risk indicators. Changes in the viral load observed during the different stages of the illness were significantly different in both groups (Figure 5). Interestingly, we observed that the viral load remained elevated in all the disease phases in patients with SD, both on days 8–10 from the onset of symptoms, and in the period after defervescence (Figure 4). Conversely, viral load decreased in patients without SD during the same stages. Our results showed that the high levels of viremia in the acute phase in patients with SD are elevated during the critical and convalescent phases. However, in cases of D ± WS, there was a significant clearance of viral load after the acute phase. Our data are consistent with those from other studies showing a positive correlation between viremia load and disease severity performed in the framework of the 1997 WHO scheme [12,27,28,31,32]. Patients with a high viremia level of >3.5 Log_10_ copies/μL (OR, 3.36; 95% CI, 1.58–7.11) during the defervescence stage had the highest risk of SD (OR, 7.11; 95% CI, 2.41–20.9). Similarly, a platelet count of <35,000 μL was considered a valuable predictive variable for differentiating the severity of illness. SD patients were associated with a risk 2.84 higher to present very low platelet levels (Table 6). We found that patients who presented a secondary DENV infection were associated with a higher risk of developing SD than did those with a primary infection (OR, 5.67; 95% CI, 1.22–26.22). Given that four predictive variables were used together in the risk analysis, it is important that such variables be combined to increase the efficiency and strength of this model for prognosis on the disease severity.

Logistic regression analysis was also conducted utilizing data obtained following the WHO 1997 classification. The same predictor variables obtained in the correspondence analysis were evaluated. Surprisingly, it was not possible to identify any useful risk factor (Table 7) to discriminate between patients with FD and DHF (I–II) or with DHF (III–IV), even though the model determined that variables such as stage of illness, infection type, and platelet count showed significance (p < 0.05).

The increased sensitivity of the 2009 WHO classification in identifying severe cases [7,17,30,33] theoretically implies an increase in hospital admissions, overstretching the capabilities of health services [17,34]. Interestingly, we found that patient hospital admissions were infrequent, and benefits exceeded costs. The CBR of both WHO classifications showed that the 2009 WHO scheme displayed greater benefit (537%) and reduced cost (10.2%) when compared with the 1997 WHO scheme. Consequently, the CBR was also low (0.019). Given the absence of data from the Americas regarding the evaluation of CBR, in our study, the CBR was evaluated in reference on a study conducted in children in Thailand [34]. In our analysis, the benefit (537%) was greater than those performed in Thailand (32.5%); furthermore, in the present study the cost (10.2%) was lower than the Thailand study (101%). Consequently, the overall CBR in the current study (0.019) was lower than the Thailand report (3.1). The difference might be explained by the populations evaluated in Kalayanarooj’s report [34] that includes only children, whereas we included both, adults and children.

An early diagnosis of SD and timely treatment could help health services avoid overstretching hospital and health center capabilities. To our knowledge, this is the first study demonstrating associations between viral load and secondary infection among patients with SD by using the 2009 WHO classification. This scheme is an appropriate tool for the early diagnosis and opportune treatment of SD patients, and used for evaluating factors associated with the progression of disease severity.

## Conclusions

The final dengue clinical phenotype in terms of disease severity was influenced by host (secondary response) and viral factors (viral load). These findings were supported by a logistic regression analysis that demonstrated predictive or prognostic values within the D/SD scheme classification. However, contrary results were obtained when we applied the classification by category (WHO 1997), where it was not possible to differentiate risk factors between patients with DF or with DHF/DSS. Clinically, a critical stage in determining the progression to SD is the phase of transition from fever to defervescence, in which hemorrhage, thrombocytopenia, plasma leakage and/or circulatory failure might occur. This is a critical period for determining the severity of the illness. The 2009 WHO classification showed greater sensitivity than the 1997 classification for identifying severe cases. In the present study, we demonstrated that the 2009 WHO classification showed low cost and high benefit by CBR analysis. Amongst warning signs proposed by the 2009 WHO classification, we found support for abdominal pain, persistent vomiting and mucosal bleeding. Likewise, by applying the 2009 classification, all cases of DHF (grades III–IV) remained in the SD group, and the grade of DF was strongly associated with the likelihood of D–WS. Timely identification of SD enables accurate early decisions, allowing proper management of health resources for the benefit of patients at risk for SD. This is possible based on the 2009 WHO classification.
